# Cross-Sectional Associations between Living and Built Environments and Depression Symptoms among Chinese Older Adults

**DOI:** 10.3390/ijerph19105819

**Published:** 2022-05-10

**Authors:** Fangfang Hou, Xiao Han, Qiong Wang, Shuai Zhou, Jingya Zhang, Guodong Shen, Yan Zhang

**Affiliations:** 1 School of Health Service Management, Anhui Medical University, Hefei 230032, China; 2045010499@stu.ahmu.edu.cn (F.H.); 2045010498@stu.ahmu.edu.cn (X.H.); 2145010558@stu.ahmu.edu.cn (Q.W.); 2145010573@stu.ahmu.edu.cn (S.Z.); 1813044009@stu.ahmu.edu.cn (J.Z.); 2 Department of Geriatrics, The First Affiliated Hospital of University of Science and Technology of China, Gerontology Institute of Anhui Province, Division of Life Sciences and Medicine, University of Science and Technology of China, Hefei 230001, China; 3 Anhui Provincial Key Laboratory of Tumor Immunotherapy and Nutrition Therapy, Hefei 230001, China

**Keywords:** depression symptoms, environment, older adults

## Abstract

In this study, we explored the cross-sectional associations between living and built environments and depression among older Chinese adults. Data from 5822 participants were obtained. Depression symptoms were evaluated through the use of the Patient Health Questionnaire (PHQ-9), with a score higher than 4 categorized as having depression symptoms. The living environment was assessed by asking about dust in the environment and barrier-free facilities. We considered the presence of amenities within a 10 min walking distance and the proportion of green space within an 800 m distance from participants’ dwellings to reflect the built environment. Data were analyzed by multilevel logistic regression. Participants living in a non-dusty environment with proximity to green space had a lower risk of depression (non-dusty environment: OR = 0.784, 95% CI = 0.642, 0.956; green space: OR = 0.834, 95% CI = 0.697, 0.998). However, having no access to barrier-free facilities and hospital proximity increased the depression risk (barrier-free facilities: OR = 1.253, 95% CI = 1.078, 1.457; hospital: OR = 1.318, 95% CI = 1.104, 1.574). Dusty environments, access to barrier-free facilities and proximity to hospitals and green spaces were associated with depression symptoms among older Chinese adults.

## 1. Introduction

Depression is an increasingly common mental disorder that not only endangers both individuals’ physical and psychological health, but also places a burden on their families and society. In particular, the COVID-19 pandemic led to a dramatic increase in the number of people with depression. It has been estimated that approximately 246 million people are currently affected by depression worldwide [1]. Depression leads to considerable disease burden and premature mortality, and is predicted to be the leading cause of death worldwide in 2030 [2].

Older adults are particularly vulnerable to depression symptoms, especially those with chronic illness and cognitive impairment [3,4,5]. Both age-related and disease-related processes, such as endocrine and immune changes, together with psychological changes [6,7,8,9,10], increase vulnerability to depression or trigger depression in people already at risk, leading to emotional suffering, disability, suicide, increased mortality and other poor health outcomes in older adults [11,12]. Many recent studies have also suggested potential roles of environmental factors in depression symptoms among older adults [13,14,15,16]. Living environment, that is, an individuals’ surroundings [17] (e.g., noise pollution), might have some social psychological effects that trigger depression [18,19]. The built environment (constructed artificial structures, e.g., streets and buildings) [20] might also play a role in inducing depression; for example, a study conducted in sub-Saharan Africa revealed that the availability of green areas was associated with lower rates of incident depression [21]. Another study indicated that individuals living on an aesthetically pleasing street and with fewer security features might have a lower likelihood of depression [22]. Although environmental factors have been recommended to be included in geriatric depression risk estimation [13], studies addressing the relationships between environmental factors and depression symptoms among older Chinese adults remain insufficient.

The purpose of the present study was to investigate the cross-sectional associations between living and built environments and depression symptoms in older Chinese adults. Considering the accelerating aging process and the high prevalence of geriatric depression in China [23], the results of the current study might have implications for public health officials by informing age-friendly community design and construction [24] to facilitate the maintenance of mental well-being among older adults.

## 2. Methods

### 2.1. Study Design

The data used in the present study were obtained from the Anhui Healthy Longevity Survey (AHLS). The details of the AHLS have been reported elsewhere [25]. Briefly, the AHLS is an ongoing study that aims to investigate major noncommunicable disease prevention and control involving behavioral intervention strategies among older adults (60 years old or older) in Anhui Province, located in eastern China. A multistage sampling strategy was applied to collect representative samples. Four cities (Fuyang, Chuzhou, Lu’an and Xuancheng) were first purposively selected to represent the northern, eastern, western and southern areas of Anhui. Then, 3 to 5 neighborhoods or villages were purposively sampled in each selected city. Finally, the older inhabitants (60 years old and older) in the selected urban or rural communities who had normal communication ability were invited to participate in the study. The recruitment of the participants stopped when the number of participants in each city reached approximately 1500, with half of the participants being from urban neighborhoods and half being from rural villages. The total sample size was 6211, and the data collection process was initiated in July 2019 and lasted approximately two months.

### 2.2. Participants

Participants were excluded if they (1) failed to complete the depression evaluation (*n* = 43) or (2) had any missing value of the covariates (*n* = 346). A total of 5822 (93.7%) participants were eligible and included in the final data analysis. It was emphasized that participation was voluntary, and informed consent was obtained prior to study enrollment. Ethical approval was received from the ethics committee of Anhui Medical University (approval no. 2020H011).

### 2.3. Depression Symptom Assessment

The Patient Health Questionnaire (PHQ-9) was applied as the depression symptom screening instrument for the participants in the current study. The PHQ-9 is a widely used instrument that asks about the frequency of the presence of significant depressive symptoms in the two weeks before the survey. A previous study concluded that the PHQ-9 had acceptable psychometric properties for depression screening among older Chinese individuals [26]. The PHQ-9 has 9 items, and a higher score reflects more severe depression symptoms (maximum total score is 30). In this study, the participants were categorized into “nondepressed” (0–4) and “depressed” (5–30) [27,28] groups based on the total PHQ-9 scores.

### 2.4. Assessment of Living Environment and Built Environment

The assessment of the living environment of the participants included exposure to uncomfortable and interfering environmental dust in their daily lives. The participants were asked, “Are you exposed to dust in your daily life?” [29,30,31]. Moreover, the participants were asked whether there were barrier-free facilities (e.g., ramps and elevators) in the places that they frequently visited.

The built environment of the participants was reflected by the presence of the four daily life-related amenities—supermarkets, hospitals, restaurants and parks—within a 10 min walking distance (i.e., 800 m) from their dwellings. First, the latitude and longitude coordinates of the participants’ homes were obtained by the online extraction map tool available from https://www.17ditu.com (accessed on 15 December 2020). Second, restaurants, parks, hospitals and supermarkets were designated as points of interest, and the locations of the abovementioned amenities were targeted by using the Baidu Map (https://lbsyun.baidu.com/index.php?title=jspopular, accessed on 17 January 2021). Restaurants, parks, hospitals and supermarkets were selected because they may help inhabitants with social contact, physical exercise, medication and day-to-day activities in China. Third, based on the coordinates, the distances between the homes and the abovementioned amenities were determined. According to the estimation that an older adult can walk approximately 800 m in 10 min, the four amenities were labeled “any” when the calculated distances between the dwellings and the amenities were less than 800 m and “none” if such distances exceeded 800 m.

As green space has been suggested to have potential effects on individuals’ mental well-being [21], the percentages of green spaces (e.g., natural forest) within 800 m from the participants’ dwellings (800 m buffer) were also calculated. The green space was first discriminated by land use layer information obtained from the Open Street Map (https://www.openstreetmap.org, accessed on 17 January 2021), followed by the percentage computation conducted using ArcGIS 10.6. Green space percentages were categorized into “any” and “none” due to the extremely skewed distribution.

### 2.5. Covariates

Participants’ basic characteristics, health-related behaviors and chronic conditions were treated as covariates in the analysis. Age data were reported by the participants themselves and were introduced into the models continuously. Education level was divided into low (illiterate), medium (formally educated for 0–6 years) and high (formally educated for over 6 years). Participants were divided into two groups (married or unmarried) according to their marital status. Annual income was set as an indicator of economic status and was classified into two categories (below 6500 RMB and 6500 RMB and above).

Health-related behaviors that might be related to depression (smoking, drinking, self-rated sleeping quality [32] and physical inactivity [33,34,35,36]) and chronic conditions (hypertension and diabetes [37,38,39]) were also treated as covariates. Regarding smoking status, participants were classified into two groups based on whether they were current smokers at the time of the survey (current smoker and noncurrent smoker). The participants were categorized into three groups by drinking status (never, former and current). Self-reported sleeping quality over the past month was divided into “very good”, “good”, “bad” and “very bad”. Physical inactivity status was reflected by daily sedentary hours, and the continuous data were self-reported by the participants. The participants’ height and weight were measured, and body mass index (BMI) was calculated based on the formula BMI = weight (kg)/height (m)^2^. Overweight was defined as a BMI of 24 or higher according to the Chinese criterion [40].

For the chronic conditions, the participants were asked, “Have you ever been formally diagnosed with hypertension/diabetes by the registered physicians in a hospital above the county level?” Then, the participants were classified into two groups according to their responses (yes or no).

### 2.6. Statistical Analysis

A multilevel regression was fitted to model the associations between living and built environments and the presence of depression symptoms due to the possible nested data structures; for example, participants living in the same community may share similar environmental features. Specifically, a multilevel data structure, which comprised 5822 individuals (at level 1) nested within 21 urban or rural communities (at level 2), was used for the data analysis. Data distributions of PHQ-9 scores were checked for normality prior to the data analysis. However, the PHQ-9 scores were skewed distributed and could not be adjusted for normality by data transformation. Thus, multilevel logistic regression strategies were adopted, with depression status (nondepressed or depressed) introduced as the dependent variable. We introduced living environment factors (exposure to a dusty environment and the presence of nearby barrier-free facilities) and built environmental factors (the presence of restaurants, parks, hospitals and supermarkets within a 10 min walking distance and the presence of green spaces within an 800 m buffer) into regression models to test their potential impacts. Analyses stratified by sex were also conducted [41,42,43].

Sensitivity analyses were conducted to test the robustness of the results. Additional analyses were performed among the participants who were overweight (*n* = 2938) and those who self-reported living with hypertension (*n* = 2918).

Data analyses were performed with Stata 15.0. Figures were drawn using Microsoft Excel (Microsoft, Redmond, WA, USA) and SPSS 24.0 (IBM Corporation, Armonk, NY, USA) for Windows. The statistical significance level was set at *p* < 0.05.

## 3. Results

Table 1 presents the basic characteristics of the participants in the study. The proportion of females was slightly higher than that of males (45.6% and 54.4% for males and females, respectively). The mean age of the participants was 71.0 years. Almost half of the participants (49.1%) were illiterate. More than half of the participants were overweight (50.5%) or had high blood pressure (50.1%). A total of 9.8% of the participants reported having exposure to a dusty environment, and a majority of participants (63.4%) reported having no barrier-free facilities nearby. About 39.1% of the participants were categorized as having depression symptoms.

**Figure 1:**
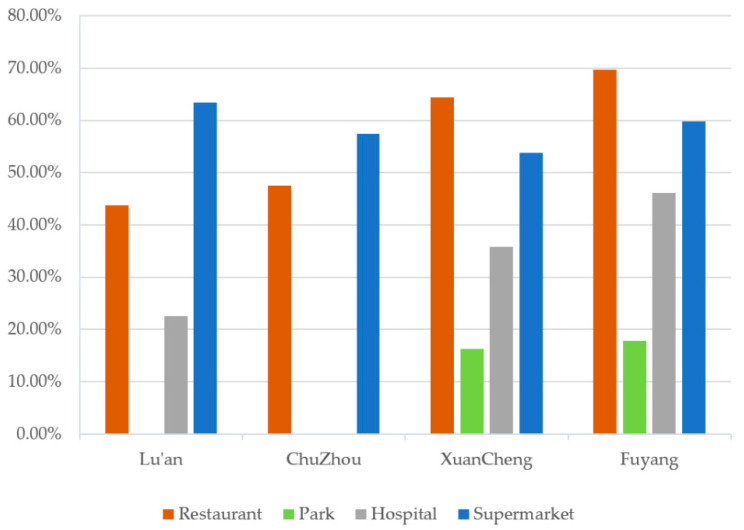
Proportions of people with restaurants, parks, hospitals and supermarkets within an 800 m buffer by city (%).

**Figure 2:**
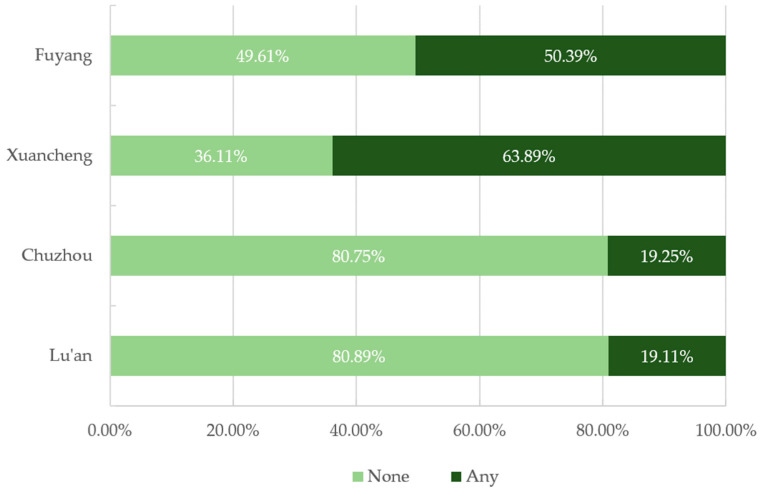
Proportions of the people with and without green space within an 800 m buffer by city (%).

Figure 1 shows the proportions of the participants with four daily life-related amenities (supermarkets, parks, restaurants and hospitals) within a 10 min walking distance (800 m) in the study area. Regarding the four amenities, over 50% of the participants had supermarkets within a 10 min walking distance from their homes in all four selected cities; however, the proportions of participants with parks within this distance were very low in Lu’an and Chuzhou. The proportions of individuals with green space within an 800 m buffer were also relatively low in Lu’an (19.11%) and Chuzhou (19.25%) (Figure 2).

The associations between living and built environments and depression symptoms are shown in Table 2. Participants living in a non-dusty environment had a lower risk of depression (OR = 0.767, 95% CI: 0.630, 0.934), although such association existed only among females (OR = 0.732, 95% CI: 0.560, 0.957) and not males. The participants with no access to barrier-free facilities had a higher risk of depression (OR = 1.251, 95% CI: 1.077, 1.453); barrier-free facilities were not associated with depression status among females. General weak and sex-specific associations between built environments and depression symptoms were detected. Male participants who had a park within a 10 min walking distance had a lower risk of depression (OR = 0.631, 95% CI: 0.412, 0.965). Participants who had a hospital within a 10 min walking distance had an elevated risk of depression (OR = 1.347, 95% CI: 1.139, 1.594), and this association seemed to be stronger among females. Participants who had any green space within an 800 m buffer around their homes tended to have a decreased risk of depression (OR = 0.752, 95% CI: 0.632, 0.896), although such a significant association was not found among males.

The sex-specific associations between multiple environmental factors and depression symptoms are presented in Table 3. Participants living in a non-dusty environment had a lower risk of depression (OR = 0.784, 95% CI: 0.642, 0.956). Participants who had no access to barrier-free facilities had a higher risk of depression (OR = 1.235, 95% CI: 1.078, 1.457). Living in proximity to a hospital was associated with higher depression risk (OR = 1.318, 95% CI: 1.104, 1.574). Participants who had any green space within an 800 m buffer around their homes had a lower risk of depression (OR = 0.834, 95% CI: 0.697, 0.998), but this factor was only associated with a decreased likelihood of depression among females (OR = 0.768, 95% CI: 0.603, 0.978).

The sensitivity analyses conducted among overweight participants revealed similar results to the main analyses. Overweight participants living in a non-dusty environment had a lower risk of depression (OR = 0.776, 95% CI: 0.636, 0.947), although such association only remained significant among females. Overweight participants who had no access to barrier-free facilities had a higher risk of depression (OR = 1.246, 95% CI: 1.072, 1.449). However, living in proximity to a hospital was associated with an elevated risk of depression (OR = 1.341, 95% CI: 1.123, 1.601). The sensitivity analyses that were restricted to participants with hypertension also yielded similar results. Hypertensive participants living in a non-dusty environment had a lower risk of depression (OR = 0.785, 95% CI: 0.644, 0.958). Hypertensive participants who had no access to barrier-free facilities had a higher risk of depression (OR = 1.254, 95% CI: 1.078, 1.458). However, the hypertensive participants living near a hospital had a higher depression risk (OR = 1.318, 95% CI: 1.103, 1.573).

## 4. Discussion

In this study, we explored the underlying roles of living and built environments in the presence of depression symptoms among older adults living in Anhui, a province located in eastern China. Our results indicated that living and built environments were associated with the presence of depression symptoms among older adults; however, this influence seems to be affected by sex. The sex differences might be partially explained by the relatively high vulnerability of females [44,45], although the exact mechanisms still need further investigation. This study was the first to date to assess the impact of environmental factors on depression symptoms among older adults living in China.

The literature suggests that some environmental factors might be linked to the occurrence of mental disorders via biological pathways (i.e., dysfunction of the hypothalamic-pituitary axis [46] or neurotransmitter [47]). Similarly to many previous studies [48,49,50], we found that participants living in a non-dusty living environment were less likely to be depressed. Particulate matter in home dust might cause neurotransmitter dysfunction, leading to oxygen deficiency that results in alterations in neural systems that increase the risk of depression [51,52]. The linkage of a dusty environment and depression symptoms might also be explained by poor visibility, a contaminated environment and increased respiratory disease caused by a dusty environment [31]. Environmental barriers, either at home or in the community, cause more restrictions and inconveniences in the daily lives of elderly individuals, especially those with functional limitations [53]. Negative emotions, such as stress and feelings of isolation generated from long-term inconvenience, could contribute to inducing depression [54,55]. The outcomes of the current study highlight the significance of environmental modifications, including improving barrier-free facilities both at home and in the community, in mental well-being maintenance and healthy aging [56,57].

A built environment that supports healthy behaviors might help reduce the risk of depression because healthy behaviors, such as physical activity and social interaction, are helpful for stress relief and improving mood [58,59,60] and, in turn, reduce the risk of depression. In this study, participants who had green space within 800 m were less likely to have depression symptoms, which could be explained by the promotion of physical activity [61,62], reduced harmful exposure (i.e., noise or dust) [63,64] or restored mental capacities (i.e., stress relief) [65], although such associations were detected only in females. This finding might be attributed to sex differences in participation in physical activity and social interaction [66,67]. Interestingly, the study found that participants who had hospitals near their dwellings were more likely to have depression symptoms. High healthcare accessibility might be helpful for the early detection and standardized treatment of depression; however, significant barriers to the availability of care for depression still exist in most hospitals in China, despite the high demand for mental health services [68]. Additionally, the hospital environment might also create negative emotions (e.g., unnecessary stress, anxiety) that might generate depression in people living nearby [69,70].

This study has a few strengths. First, the data used for analysis were obtained from a large sample size with geographical and urban–rural gaps fully considered so that representativeness was guaranteed. Second, a number of covariates, including demographic and behavioral factors, were employed so that the independent roles of living environment and built environment factors could be tested; thus, the validity of the results was high. Moreover, for the measurements of the built environment, objective calculation was adopted instead of relying on self-report by participants, so the reporting bias could be somewhat reduced.

However, the study is not without limitations. First, cause and effect conclusions could not be drawn due to the cross-sectional design. Although cross-sectional associations were observed in the current study, well-designed longitudinal studies are needed to verify the effects of the environment on the development of depression among older adults in the future. Second, many cities in China are experiencing rapid and intensive urbanization, so possible massive changes in the built environment [71] might obscure the true associations. Therefore, future studies should take the changes in built environment factors into consideration to explore the true associations and provide evidence of the influence of built environment intervention on the mental health maintenance of older adults. Third, social and interpersonal environments that are related to depression occurrence were not incorporated in the present study. For deep exploration, the roles of many environmental components, such as social stress, life events and work satisfaction, should be investigated under a more comprehensive framework in the future. Finally, although the PHQ-9 has been identified as a reliable instrument for depression screening, the scale alone may not be sufficient for accurate depression diagnosis. Standard diagnostic procedures or more comprehensive instruments (e.g., the Beck Depression Inventory) should be applied in future studies to avoid possible misclassification bias, which may exist in the current study.

## 5. Conclusions

In the current study, we explored the cross-sectional associations among living environment, built environment and depression symptoms among 5822 older Chinese adults. Living in a non-dusty environment and having green space near one’s residence were associated with a reduced risk of depression, and having no access to barrier-free facilities and having hospitals nearby were associated with a higher risk of depression. Since geriatric depression is highly prevalent worldwide, our findings reveal the importance of considering the living environment and built environment in developing interventions for the maintenance of mental well-being for community-dwelling older adults. Our findings also provide novel insights for healthy aging, especially for the creation of an aging-friendly environment.

## Figures and Tables

**Table 1:** Basic characteristics of the enrolled participants.

	N	Mean/Percentage
Age	-	71.0
Sex (Male)	2653	45.6
City		
Lu’an (West)	1533	26.3
Chuzhou (East)	1527	26.2
Xuancheng (South)	1343	23.1
Fuyang (North)	1419	24.4
Urban	2876	49.4
Education level		
Low (illiterate)	2861	49.1
Medium (1–6 years)	1620	27.8
High (>6 years)	1341	23.0
Marital status		
Married	4238	72.8
Unmarried	1584	27.2
Annual income		
Less than 6500 RMB	3499	60.1
6500 RMB and above	2323	39.9
Current smoker (%)	1233	21.2
Drinking status		
Never	3331	57.2
Former	232	4.0
Current	2259	38.8
Sleeping quality		
Very good	1203	20.57
Good	3227	55.18
Bad	1180	20.18
Very bad	238	4.07
Physical inactivity		
Sitting hours	-	4.3 ^a^
Chronic conditions		
Diabetes (%)	919	15.8
Hypertension (%)	2918	50.1
Overweight (%)	2938	50.5
Living environment		
Dusty environment (yes)	572	9.8
Barrier-free facilities (no)	3690	63.4
Depression status		
Yes	2279	39.1
No	3543	60.9

^a^ Median.

**Table 2:** Independent associations between each living environmental and built environmental factor and depression, stratified by sex.

	Both Sexes (OR, 95% CI)	Male (OR, 95% CI)	Female (OR, 95% CI)
***Living environment***			
Dusty environment (Ref: yes) No	0.767 (0.630, 0.934) **	0.810 (0.602, 1.090)	0.732 (0.560, 0.957) *
Barrier-free facilities (Ref: yes) No	1.251 (1.077, 1.453) **	1.371 (1.091, 1.723) **	1.151 (0.944, 1.403)
***Built environment***			
Restaurant (Ref: none)			
Any	1.102 (0.932, 1.303)	1.053 (0.846, 1.311)	1.028 (0.824, 1.283)
Park (Ref: none)			
Any	0.832 (0.639, 1.083)	0.631 (0.412, 0.965) *	0.979 (0.700, 1.368)
Hospital (Ref: none)			
Any	1.347 (1.139, 1.594) **	1.203 (0.937, 1.545)	1.414 (1.133, 1.766) **
Supermarket (Ref: none)			
Any	1.158 (0.973, 1.378)	1.215 (0.931, 1.587)	1.005 (0.802, 1.258)
Green space within 800 m buffer (Ref: none)			
Any	0.752 (0.632, 0.896) **	0.847 (0.674, 1.064)	0.700 (0.552, 0.888) **

Adjusted for sex, age, urban/rural, city, education, marital status, annual income, diabetes, hypertension, drinking status, smoking, overweight, self-rated sleeping quality and sedentary hours. * *p* < 0.05 ** *p* < 0.01.

**Table 3:** Adjusted associations between multiple environmental factors and depression, stratified by sex.

	Both Sexes (OR, 95% CI)	Male (OR, 95% CI)	Female (OR, 95% CI)
***Living environment***			
Dusty environment (Ref: yes) No	0.784 (0.642, 0.956) *	0.814 (0.604, 1.097)	0.752 (0.575, 0.985) *
Barrier-free facilities (Ref: yes) No	1.253 (1.078, 1.457) **	1.396 (1.110, 1.756) **	1.133 (0.927, 1.384)
***Built environment***			
Park (Ref: none)			
Any	0.764 (0.583, 1.000)	0.567 (0.366, 0.879) *	0.910 (0.645, 1.285)
Hospital (Ref: none)			
Any	1.318 (1.104, 1.574) **	1.257 (0.969, 1.631)	1.344 (1.066, 1.696) *
Green space within 800 m buffer (Ref: none)			
Any	0.834 (0.697, 0.998) *	0.920 (0.728, 1.163)	0.768 (0.603, 0.978) *

Adjusted for sex, age, urban/rural, city, education, marital status, annual income, diabetes, hypertension, drinking status, smoking, overweight, self-rated sleeping quality and sedentary hours. * *p* < 0.05 ** *p* < 0.01.

## Data Availability

Data can be made available upon request.
